# Quantitative and clinical implications of the EARL2 versus EARL1 [^18^F]FDG PET-CT performance standards in head and neck squamous cell carcinoma

**DOI:** 10.1186/s13550-023-01042-w

**Published:** 2023-10-25

**Authors:** Maurice C. Cox, Tijn Jurcka, Anne I. J. Arens, Maartje C. van Rijk, Johannes H. A. M. Kaanders, Sven van den Bosch

**Affiliations:** 1 Department of Radiation Oncology, Radboud University Medical Center, 6525 GA Nijmegen, The Netherlands; 2 Department of Radiology and Nuclear Medicine, Radboud University Medical Center, Nijmegen, The Netherlands

**Keywords:** Head and neck cancer, PET-CT, EARL, SUV

## Abstract

**Background:**

The EANM Research Ltd. (EARL) guidelines give recommendations for harmonization of [^18^F]FDG PET-CT image acquisition and reconstruction, aiming to ensure reproducibility of quantitative data between PET scanners. Recent technological advancements in PET-CT imaging resulted in an updated version of the EARL guidelines (EARL2). The aim of this study is to compare quantitative [^18^F]FDG uptake metrics of the primary tumor and lymph nodes in patients with head and neck squamous cell carcinoma (HNSCC) on EARL2 versus EARL1 reconstructed images and to describe clinical implications for nodal staging and treatment.

**Methods:**

Forty-nine consecutive patients with HNSCC were included. For all, both EARL1 and EARL2 images were reconstructed from a singular [^18^F]FDG PET-CT scan. Primary tumors and non-necrotic lymph nodes ≥ 5 mm were delineated on CT-scan. In the quantitative analysis, maximum standardized uptake values (SUV_max_) and standardized uptake ratios (SUR_max_, i.e., SUV_max_ normalized to cervical spinal cord uptake) were calculated for all lesions on EARL1 and EARL2 reconstructions. Metabolic tumor volume (MTV) and total lesion glycolysis were compared between EARL1 and EARL2 using different segmentation methods (adaptive threshold; SUV2.5/3.5/4.5; SUR2.5/3.5/4.5; MAX40%/50%). In the qualitative analysis, each lymph node was scored independently by two nuclear medicine physicians on both EARL1 and EARL2 images on different occasions using a 4-point scale.

**Results:**

There was a significant increase in SUV_max_ (16.5%) and SUR_max_ (9.6%) of primary tumor and lymph nodes on EARL2 versus EARL1 imaging (*p* < 0.001). The proportional difference of both SUV_max_ and SUR_max_ between EARL2 and EARL1 decreased with increasing tumor volume (*p* < 0.001). Absolute differences in MTVs between both reconstructions were small (< 1.0 cm^3^), independent of the segmentation method. MTVs decreased on EARL2 using relative threshold methods (adaptive threshold; MAX40%/50%) and increased using static SUV or SUR thresholds. With visual scoring of lymph nodes 38% (11/29) of nodes with score 2 on EARL1 were upstaged to score 3 on EARL2, which resulted in an alteration of nodal stage in 18% (6/33) of the patients.

**Conclusions:**

Using the EARL2 method for PET image reconstruction resulted in higher SUV_max_ and SUR_max_ compared to EARL1, with nodal upstaging in a significant number of patients.

## Background

Positron emission tomography (PET) with fluor-18-fluorodeoxyglucose ([^18^F]FDG) in combination with computed tomography (CT) is increasingly utilized for radiation treatment planning in patients with head and neck squamous cell carcinoma (HNSCC). The use of PET-CT scanners with different hardware specifications or methods of image acquisition and reconstruction can result in undesired variation of quantitative [^18^F]FDG uptake metrics [1]. To ensure the reproducibility of quantitative data between PET scanners, the European Association of Nuclear Medicine (EANM) has initiated the EANM Research Ltd. (EARL) harmonization program. These give guidelines on how to perform PET imaging, aiming to harmonize patient preparation, scan acquisition, and image reconstruction [2].

The first version of the EANM guidelines (EARL1) was introduced in 2010 [3, 4]. Over the years, multiple technological advances in PET-CT imaging regarding both hard- and software have improved contrast recovery with better spatial resolution and lesion detectability [5, 6]. Among these new developments are the introduction of time-of-flight, point spread function, smaller voxel sizes and digital silicon photomultiplier detectors. An updated version of the EANM guidelines (EARL2) was introduced in 2019 to take these developments into account [6, 7]. Compared to EARL1, application of the EARL2 image reconstruction methods can result in significant changes in quantitative [^18^F]FDG uptake metrics, such as the maximum standardized uptake value (SUV_max_), SUV_mean_, metabolic target volume (MTV), and total lesion glycolysis (TLG) [8]. Changes in quantitative PET readings can have important clinical implications for tumor staging and treatment. Ly et al. reported that the use of EARL2 versus EARL1 reconstructions for lymphoma lesions led to an upgrade in Deauville score in 33% of the patients, resulting in a treatment intensification in 9% of the patients [9].

Changes of quantitative [^18^F]FDG uptake metrics as a result from EARL2 image reconstruction methods may also affect treatment of patients with HNSCC. For radiation treatment, [^18^F]FDG PET-CT imaging can be used for primary tumor segmentation and to guide dose escalation to a metabolic subvolume within the tumor [10–12]. In addition, enhanced contrast ratios with EARL2 can also improve the detection of nodal metastases and thus consequentially alter nodal staging and radiation treatment.

Therefore, the aim of this study is to compare quantitative [^18^F]FDG uptake metrics of the primary tumor and lymph nodes in patients with HNSCC using EARL2 versus EARL1 reconstructed images and to describe clinical implications for nodal staging and treatment.

## Methods

### Patient selection

This is a single center cohort study. The need for written informed consent was waived by the local ethics committee as the study was conducted retrospectively from data obtained for clinical purposes (reference number 2021-9835). A sequential cohort of 230 patients with squamous cell carcinoma of the oropharynx, hypopharynx or larynx that received definitive (chemo)radiotherapy between November 2020 and October 2021 was screened for eligibility. Acquisition of a [^18^F]FDG PET-CT scan in radiation treatment position with both EARL1 and EARL2 PET image reconstructions was mandatory. As from November 2020, acquisition of both EARL1 and EARL2 reconstructed images is standard practice at our institution. Patients with previous oncologic treatment (e.g. radiotherapy or tumor reductive surgery) of the head and neck area were excluded.

### PET-CT acquisition and reconstruction

A PET scan, a low-dose CT scan for attenuation correction and an iodine contrast enhanced diagnostic CT scan for radiation treatment planning was acquired in one session on a Biograph mCT40 PET-CT scanner (Siemens Medical Solutions, Knoxville TN, USA). Imaging was acquired in radiation treatment position, using a customized neck support (AccuForm Custom Cushions, Accuform, MEDTEC, Orange City, IA) and a five-point fixation mask for immobilization of the head, neck and shoulders (HNS Mask-Nose Hole in Efficast 2.0 mm MAXI, Orfit Masks, Orfit Industries NV, Wijnegem, Belgium). Prior to [^18^F]FDG administration, patients fasted for at least 4 h and a serum glucose level of < 11 mmol/L was mandatory. [^18^F]FDG was intravenously administrated approximately 60 min prior to the scanning procedures (dose calculated using Eq. 1) [7].

1 $$\left[ {^{18} {\text{F}}} \right]{\text{FDG}} \left( {{\text{MBq}}} \right) = \frac{{7 \left( {{\text{MBq}}*\frac{{{\text{min}}}}{{{\text{bed}}}}*\frac{1}{{{\text{kg}}}}} \right)*{\text{patient}}\; {\text{weight}} \left( {{\text{kg}}} \right)}}{{{\text{emission}}\; {\text{acquisition}}\; {\text{duration}}\; {\text{per}}\; {\text{bed}}\; {\text{position}}\; \left( {\frac{{{\text{min}}}}{{{\text{bed}}}}} \right)}}.$$

All patients were scanned from the lower border of the clavicle to the cranium. The acquisition time was 3 min per bed position with an overlap of 43% between bed positions. The slice thickness of the CT-scan was 3 mm. EARL1 images were reconstructed with an ordered-subsets expectation–maximization (OSEM) algorithm including point spread function and time-of-flight, Gaussian Filter 7.5 mm full width at half maximum, image matrix 256*256 and voxel size 4.1*4.1*5.0 mm. EARL2 images were reconstructed with point spread function and time-of-flight OSEM, Gaussian Filter 4.3 mm, image matrix 400*400 and voxel size 2.0*2.0*5.0 mm.

### Primary tumor

For all patients, [^18^F]FDG PET-CT scans were imported into the radiation treatment planning system Pinnacle version 3.2.0.27 (Philips Medical Systems, Fitchburg, MA, USA). Primary tumors were delineated on the CT-scan based on information gathered from physical examination and diagnostic imaging. The volume and the maximum [^18^F]FDG uptake was determined in terms of SUV_max_ and maximum standardized uptake ratio (SUR_max_) on both EARL reconstructions for each delineated tumor. The tumor to cervical spinal cord standardized uptake ratio (SUR) has been shown to improve the reproducibility of quantitative [^18^F]FDG-PET data in a multicenter setting compared to SUV based approaches [13]. SUV_max_ and SUR_max_ were calculated using Eqs. 2 and 3.

2 $${\text{SUV}}_{{{\text{max}}}} \left( {\frac{{\text{g}}}{{{\text{cm}}^{3} }}} \right) = \frac{{{\text{Maximum}}\; {\text{activity}} \;{\text{concentration}} \left( {\frac{{{\text{Mbq}}}}{{{\text{cm}}^{3} }}} \right)}}{{{\text{Injected}}\; {\text{dose}} \left( {{\text{MBq}}} \right)*2^{{\frac{{ - {\text{time}} \;{\text{between}} \;{\text{injection}} \;{\text{and}}\; {\text{start}} \;{\text{scan}}\; \left( {\text{s}} \right)}}{{{\text{Half}}\; {\text{life}}\; {\text{of}} \;{\text{radionuclide}} \left( {\text{s}} \right)}}}} }}*{\text{Body}}\; {\text{weight}} \left( {\text{g}} \right)$$

3 $${\text{SUR}}_{{{\text{max}}}} = \frac{{{\text{SUV}}_{{{\text{max}}}} \text{of}}\; {\text{lesion}}\; \left( {\frac{{\text{g}}}{{{\text{cm}}^{3} }}} \right)}{{{\text{SUV}}_{{{\text{mean}}}} \;{\text{of}}\; {\text{cervical}} \;{\text{spinal}} \;{\text{cord}} \left( {\frac{{\text{g}}}{{{\text{cm}}^{3} }}} \right)}}$$

Three different segmentation methods were used to determine MTVs on both EARL1 and EARL2 reconstructed images. Delineation of MTVs was performed automatically with customized Pinnacle scripting. Thresholds used were (1) an adaptive threshold as a percentage of SUR_max_ (threshold = 116.93 * SUR_max_^−0.75^) [13], (2) a percentage of maximum [^18^F]FDG uptake (MAX40% and MAX50%) and (3) a static threshold of SUV or SUR (2.5, 3.5 and 4.5). For the SUV based segmentation methods, TLGs are calculated using Eq. 4.

4 $${\text{TLG}} \left( {\text{g}} \right) = {\text{MTV}} \; \left( {{\text{cm}}^{3} } \right)*{\text{SUV}}_{{{\text{mean}}}} \;{\text{of}} \;{\text{MTV}} \left( {\frac{{\text{g}}}{{{\text{cm}}^{3} }}} \right).$$

The method of classification errors (CE) was used to evaluate spatial overlap of the MTVs based on EARL1 and EARL2 reconstructed images [14]. An important advantage of the CE method is that it does not only take volume into account, but also the spatial position and shape of the contours due to both false-negative and false-positive volumes. The CE can range from 0 to infinite, in which a lower CE implies better spatial overlap, and is calculated using Eq. 5.

5 $${\text{Classification}}\; {\text{Error}} = \frac{{{\text{false}}\; {\text{negative}}\; {\text{volume}}\; \left( {{\text{cm}}^{3} } \right) + {\text{false}} \;{\text{positive}}\; {\text{volume}} \;\left( {{\text{cm}}^{3} } \right)}}{{{\text{Volume}}\; {\text{of}}\; {\text{the}} \;{\text{EARL}}1\, {\text{reconstruction}}\; \left( {{\text{cm}}^{3} } \right)}}$$

The false-negative volume is defined as MTV that is delineated on EARL1 but not on EARL2, and vice versa for the false-positive volume.

### Lymph nodes

All lymph nodes having a short-axis diameter of ≥ 5 mm in the axial plane were manually delineated on the CT-scan. This threshold was chosen because histopathological validation studies suggest that nodal metastases of this size can be detected by [^18^F]FDG PET-CT [15, 16]. Necrotic lymph nodes with irrefutably disturbed [^18^F]FDG distribution were not considered. Short-axis diameters, nodal volumes and quantitative [^18^F]FDG uptake parameters (i.e., SUV_max_ and SUR_max_) on both EARL1 and EARL2 reconstructed images were determined for each node.

For the qualitative analysis of nodal [^18^F]FDG uptake, both the EARL1 and EARL2 reconstructed image series were read independently by two experienced nuclear medicine physicians (AA and MvR), specialized in head and neck cancer. Each lymph node was scored separately on EARL1 and EARL2 reconstructed images using a 4-point scale (1—definitely benign, 2—probably benign, 3—probably malignant, 4—definitely malignant). To minimize observer recall bias, the time between scoring EARL1 and EARL2 reconstructed images was at least 4 weeks. Discrepancies between observers involving scores ‘3—probably malignant’ or ‘4—definitely malignant’ were resolved by consensus. Visual scores on EARL1 and EARL2 images were compared to identify consequences for radiation treatment and for N-classification (8th edition of UICC TNM classification) [17]. A change from score 1 or 2 to score 3 or 4 or vice versa was assumed to have consequence for staging and treatment.

### Statistics

All statistical analyses were performed using SPSS version 26 (IBM Corporation, New York, NY, USA). Statistical significance level was set to *p* < 0.05. Normal distribution of data was tested using the Shapiro–Wilk test. Data characterized by normal distribution were presented as mean with 95% confidence interval (95% CI) and parameters not normally distributed as median with the interquartile range (IQR). Scatter plots and intraclass correlation coefficients (with a two-way mixed model testing absolute agreement) were used to describe the relationship of maximum [^18^F]FDG uptake (i.e., SUV_max_ and SUR_max_) on EARL1 and EARL2 reconstructed imaging. To evaluate the magnitude of differences between EARL1 and EARL2, the relative differences in SUV_max_ or SUR_max_ were plotted against the average SUV_max_ or SUR_max_ on both EARL reconstructions, according to the Bland–Altman method. Mean/median differences in quantitative metrics between both EARL reconstructions were calculated based on the differences of paired data. Comparison of means between groups was done using the Student *T* test for paired data in case of a normal distribution and the Wilcoxon signed rank test for data not normally distributed. In the qualitative nodal evaluation, agreement between observers was calculated by the kappa statistic. The kappa score can range between 0 and 1, with a score of 0.00–0.20 indicating none to slight interobserver agreement, 0.21–0.40 fair, 0.41–0.60 moderate, 0.61–0.80 substantial, and 0.81–1.00 almost perfect agreement [18].

## Results

A total of 49 patients met all the in- and exclusion criteria. Patient and treatment characteristics are listed in Table 1. The mean administered dose of [^18^F]FDG was 178 MBq (95% CI 95–261). The mean time between FDG administration and image acquisition was 69 min (95% CI 55–83). The median blood glucose level was 5.7 mmol/L (IQR 5.2–6.2) at the time of [^18^F]FDG administration.

**Table 1:** Patient and treatment characteristics

	(*N* = 49)
Sex	
Male	39 (80%)
Female	10 (20%)
Age at diagnosis (years)	
Median	66
Range	52–91
T-stage	
T1	3 (6%)
T2	19 (39%)
T3	17 (35%)
T4	10 (20%)
Subsite primary tumor	
Oropharynx	24 (49%)
Larynx	17 (35%)
Hypopharynx	8 (16%)
HPV status (oropharynx)	
Negative	14 (58%)
Positive	10 (42%)
N-stage	
N0	22 (45%)
N1	11 (22%)
N2	13 (27%)
N3	3 (6%)
Delineated lymph nodes per neck level	
Level 1A	6 (3%)
Level 1B	33 (19%)
Level 2	98 (56%)
Level 3	27 (16%)
Level 4	5 (3%)
Level 5	6 (3%)
Treatment	
Radiotherapy	30 (61%)
Chemoradiotherapy	19 (39%)

### Primary tumor

**Fig. 1:**
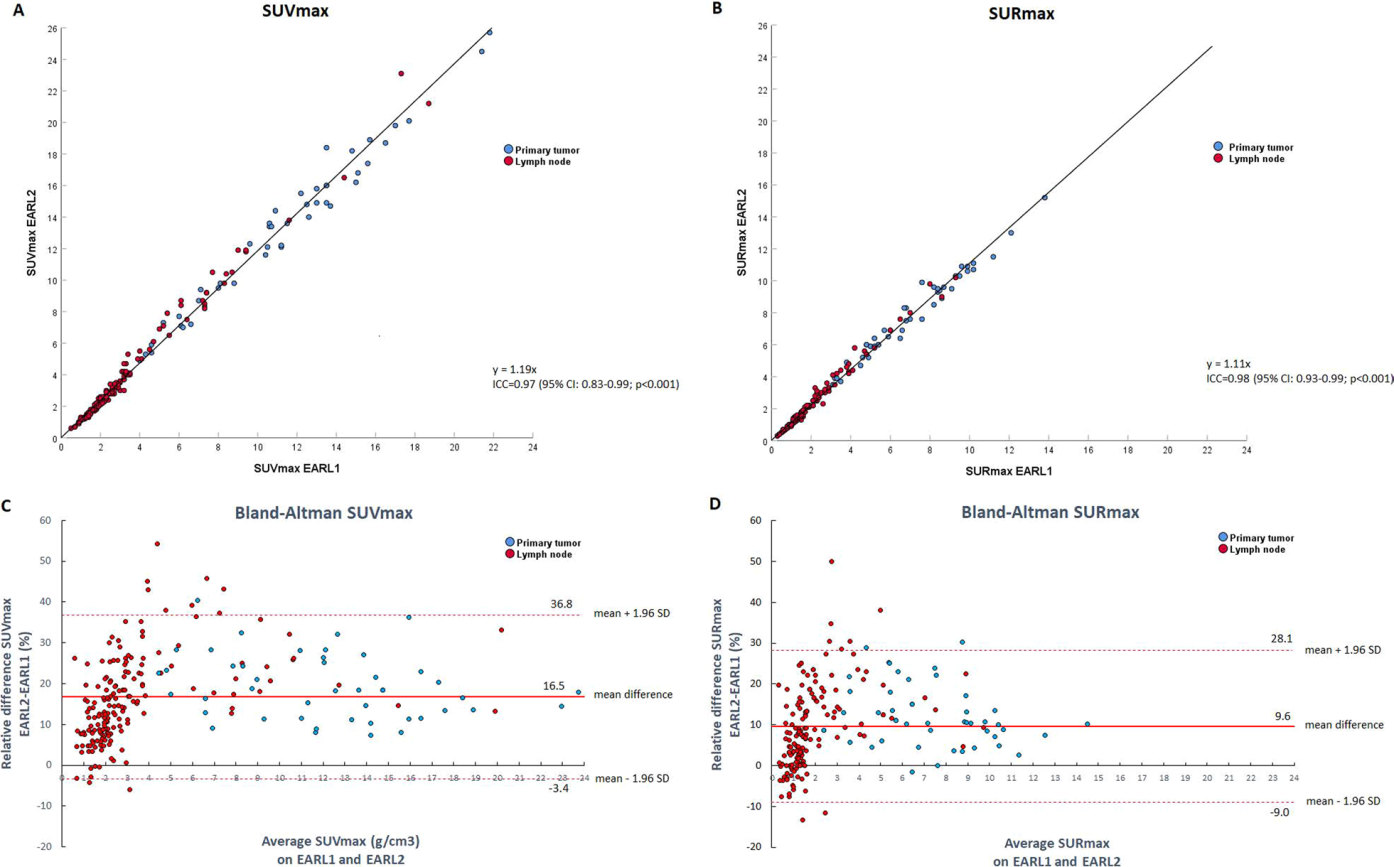
Scatter plots and Bland–Altman plots of SUV_max_ and SUR_max_. **A** and **B** Scatter plots demonstrating the relationship of SUV_max_ (**A**) and SUR_max_ (**B**) between EARL1 and EARL2. Abbreviations: ICC: intraclass correlation coefficient. **C** and **D** Bland–Altman plots demonstrating the relative differences of SUV_max_ (**C**) and SUR_max_ (**D**) on EARL2 versus EARL1

**Fig. 2:**
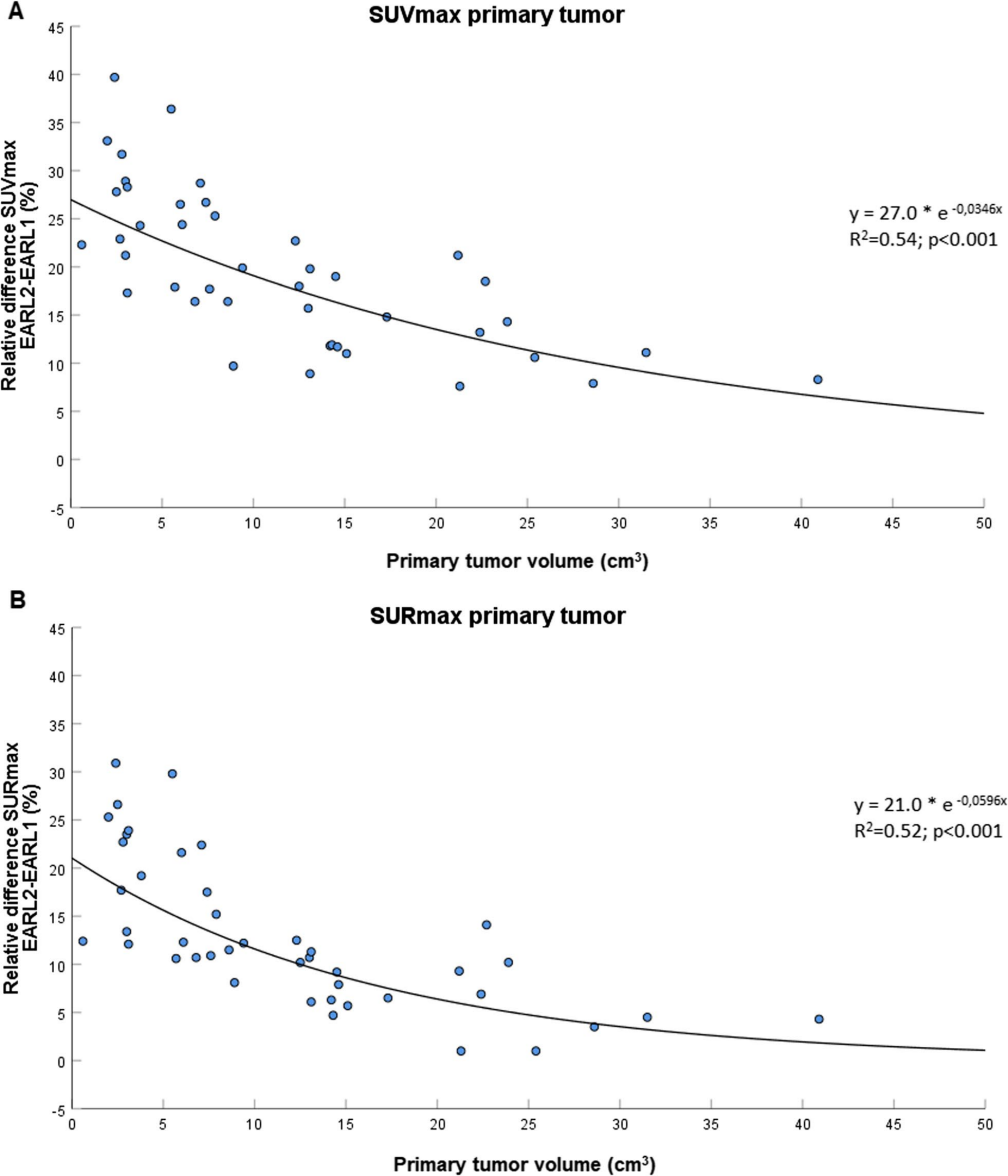
Relative difference in SUV_max_ and SUR_max_ in relation to primary tumor volume. Scatter plot showing the relative difference in SUV_max_ (**A**) and SUR_max_ (**B**) between EARL1 and EARL2, in relation to the primary tumor volume

For this analysis, four patients were excluded because tumor segmentation was not possible as a result of insufficient [^18^F]FDG uptake in the tumor (*n* = 1), or overlapping [^18^F]FDG uptake of the tumor with adjacent nodal metastases (*n* = 3). Thus, for the primary tumor analysis, 45 of 49 patients were evaluable. The median primary tumor volume as delineated on CT was 9.4 cm^3^ (IQR 5.5–17.3). The mean SUV_max_ of the primary tumor was 11.2 (95% CI 2.6–19.7) and 13.2 (95% CI 3.5–22.9) on EARL1 imaging and EARL2 imaging, respectively. The SUR_max_ was 7.0 (95% CI 1.9–12.1) on EARL1 and 7.8 (95% CI 2.4–13.2) on EARL2. For all lesions combined (primary tumors and lymph nodes), there was a strong linear relationship between maximum [^18^F]FDG uptake (i.e., SUV_max_ and SUR_max_) on EARL1 and EARL2 imaging, with an intraclass correlation coefficient of 0.97 (95% CI 0.83–0.99; *p* < 0.001) for SUV_max_ and 0.98 (95% CI 0.93–0.99; *p* < 0.001) for SUR_max_ (Fig. 1A–B). Bland–Altman plots showed a mean increase in SUV_max_ of 16.5% (95% CI − 3.4 to 36.8; *p* < 0.001) and SUR_max_ of 9.6% (95% CI − 9.0–28.1; *p* < 0.001) on EARL2 reconstructed imaging compared to EARL1 (Fig. 1C, D). The proportional difference of both SUV_max_ and SUR_max_ between EARL1 and EARL2 decreased with increasing tumor volume (Fig. 2).

MTVs and TLGs of the primary tumors for EARL1 and EARL2 reconstructed images for the various segmentation methods are shown in Tables 2 and 3, respectively. For most segmentation methods (7/9) there was a difference of the MTV on EARL2 compared to EARL1, with MTVs being significantly larger on EARL2 for 6/9 methods. Relative differences in MTVs were small using the adaptive threshold method and larger using static SUV or SUR thresholds. Also, relative differences were more pronounced using higher static thresholds (i.e., SUV4.5/SUR4.5) compared to lower static thresholds (SUV2.5/SUR2.5). For all SUV based segmentation methods, there was a significant difference of TLG between EARL1 and EARL2. The MAX40% segmentation method resulted in the smallest difference of TLG between EARL1 and EARL2.

**Table 2:** Differences in MTV between EARL2 versus EARL1 using multiple segmentation methods

Segmentation method for MTV	Median MTV EARL1 (cm^3^)	Median MTV EARL2 (cm^3^)	Median difference* (cm^3^)	Median difference* (%)	*p* value
Adaptive threshold	9.4 (4.8–18)	9.7 (5.0–17)	− 0.2 (− 0.6 to 0.1)	− 2.4 (− 5.7 to 0.7)	0.003
SUV2.5	14.5 (5.4–25)	14.7 (6.1–25)	0.1 (− 0.1 to 0.4)	1.6 (− 0.3 to 6.1)	0.024
SUV3.5	9.2 (3.4–18)	9.6 (3.6–18)	0.4 (0.3–0.6)	4.7 (1.7–14)	< 0.001
SUV4.5	6.0 (2.1–13)	6.8 (2.5–14)	0.7 (0.4–0.9)	11.3 (5.2–25)	< 0.001
SUR2.5	5.4 (2.6–15)	5.8 (2.4–14)	0.1 (− 0.4 to 0.2)	0.7 (− 2.8 to 11)	0.712
SUR3.5	3.4 (1.3–9.6)	3.7 (1.7–9.5)	0.2 (0.0–0.4)	9.2 (1.1–21)	0.001
SUR4.5	2.4 (0.6–6.6)	2.7 (0.9–7.2)	0.3 (0.2–0.6)	15 (8.1–43)	< 0.001
MAX40%	7.0 (3.3–12)	6.7 (2.9–10)	− 0.4 (− 0.9 to − 0.1)	− 7.5 (− 17 to − 2)	< 0.001
MAX50%	4.3 (2.0–7.6)	4.0 (1.8–7.6)	− 0.1 (− 0.4 to 0.2)	− 4.1 (− 20.1 to 2.5)	0.250

Absolute and relative differences between EARL1 and EARL2 are calculated in reference to EARL1

*Median based on the differences of paired data. Values in parentheses represent the interquartile range

**Table 3:** Differences in TLG between EARL2 versus EARL1 using multiple segmentation methods

Segmentation method for MTV	Median TLG EARL1 (g)	Median TLG EARL2 (g)	Median difference* (g)	Median difference* (%)	*p* value
SUV2.5	64.7 (22–168)	69.2 (28–175)	5.2 (3.5–7.3)	7.2 (4.7–15)	< 0.001
SUV3.5	59.4 (20–139)	67.1 (24–148)	7.2 (4.4–11)	12 (7.3–23)	< 0.001
SUV4.5	54.5 (16–121)	64.3 (24–135)	9.9 (6.0–14)	18 (12–38)	< 0.001
MAX40%	42.7 (17–94)	43.8 (17–102)	1.9 (− 0.5–7.9)	4.9 (− 2.7 – 8.3)	< 0.001
MAX50%	29.3 (11–71)	33.3 (12–83)	3.2 (− 0.4–7.0)	9.2 (− 2.5 – 14)	< 0.001

Absolute and relative differences between EARL1 and EARL2 are calculated in reference to EARL1

*Median based on the differences of paired data. Values in parentheses represent the interquartile range

The CE values for spatial overlap of MTVs based on EARL1 and EARL2 reconstructed images for the various segmentation methods were relatively small, ranging between 0.10 and 0.23 (Table 4). For the majority of segmentation methods, the false-negative volume was < 1 cm^3^ (MTV that is delineated on EARL1 but not on EARL2) and the false-positive volume < 0.5 cm^3^ (MTV delineated on EARL2 but not on EARL1).

**Table 4:** CE values indicating spatial overlap of MTVs on EARL1 and EARL2

Segmentation method	Median CE	False-negative volume (cm^3^)	False-positive volume (cm^3^)
Adaptive threshold	0.15 (0.12–0.18)	0.98 (0.51–1.60)	0.39 (0.27–0.63)
SUV2.5	0.10 (0.09–0.14)	1.05 (0.31–1.60)	0.55 (0.41–0.81)
SUV3.5	0.13 (0.08–0.17)	0.56 (0.14–1.00)	0.45 (0.35–0.68)
SUV4.5	0.14 (0.09–0.23)	0.26 (0.11–0.60)	0.58 (0.40–0.74)
SUR2.5	0.14 (0.10–0.20)	0.46 (0.18–1.10)	0.34 (0.19–0.47)
SUR3.5	0.18 (0.11–0.25)	0.27 (0.06–0.62)	0.42 (0.20–0.50)
SUR4.5	0.23 (0.13–0.38)	0.21 (0.03–0.43)	0.34 (0.21–0.59)
MAX40%	0.16 (0.12–0.20)	0.69 (0.48–1.20)	0.26 (0.10–0.39)
MAX50%	0.17 (0.13–0.21)	0.45 (0.24–0.66)	0.22 (0.07–0.47)

Values in parentheses represent the interquartile range. The false-negative volume is defined as MTV that is delineated on EARL1 but not on EARL2, and vice versa for the false-positive volume

### Lymph nodes

In total, 175 lymph nodes in the first 34 consecutive patients were delineated on the CT scans and analyzed, as this number of lymph nodes was considered to provide sufficient power for both the quantitative and qualitative analysis. One of the 34 patients was excluded because no lymph nodes with a short-axis diameter ≥ 5 mm were present. The median short-axis diameter of lymph nodes on CT was 6 mm (IQR 5–8), with a median nodal volume of 0.6 cm^3^ (IQR 0.4–1.3). The median SUV_max_ of lymph nodes was 2.1 (IQR 1.7–3.1) and 2.4 (IQR 1.8–3.7) on EARL1 and EARL2, respectively. The SUR_max_ was 1.3 (IQR 1.1–1.8) on EARL1 and 1.4 (IQR 1.1–2.2) on EARL2.

**Fig. 3:**
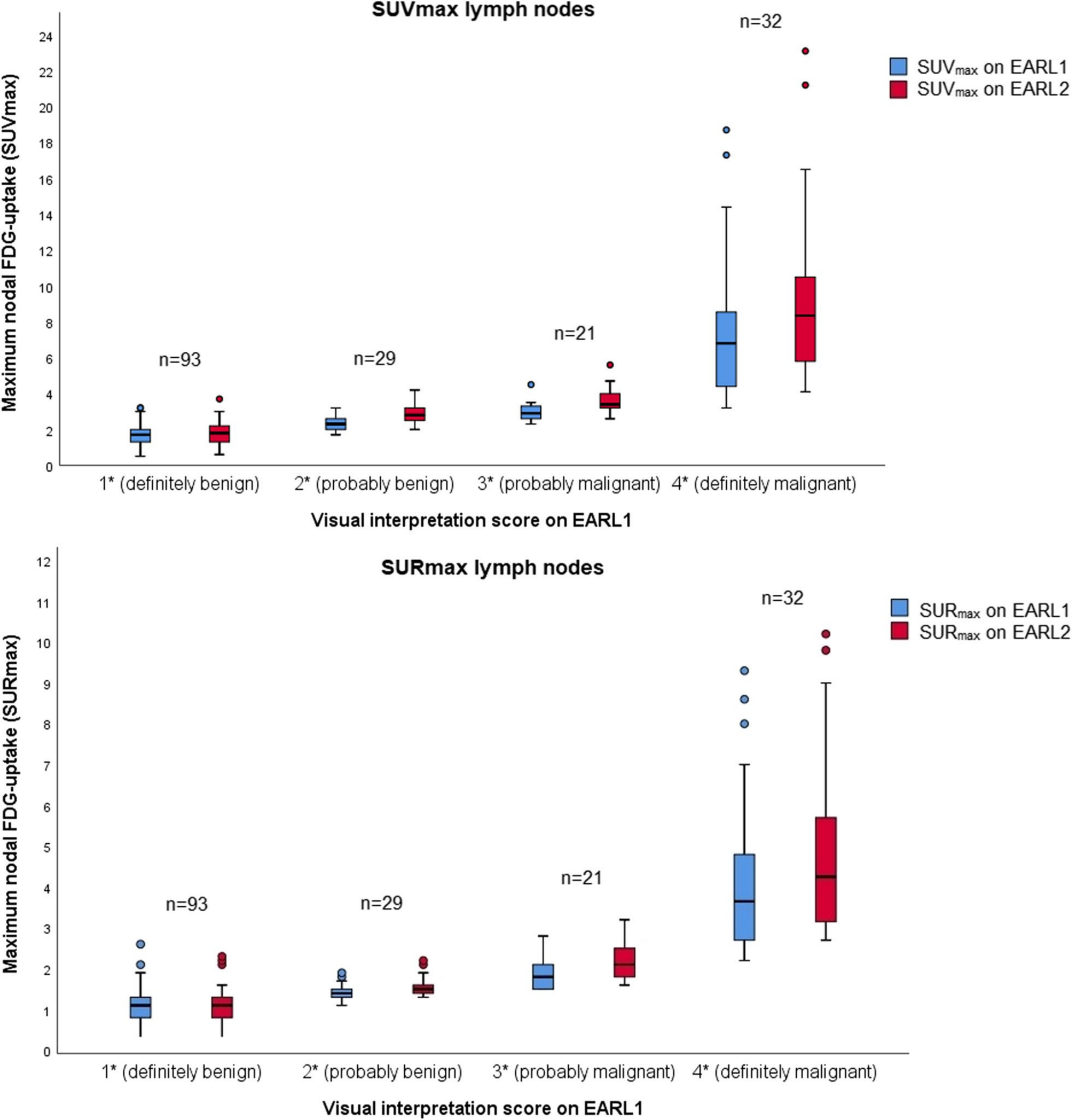
Boxplots of SUV_max_ and SUR_max_ on EARL1 and EARL2, stratified by nodal score on EARL1. Box plots showing the distribution of SUV_max_ (**A**) and SUR_max_ (**B**) of lymph nodes on EARL1 (blue) and EARL2 (red), stratified by the visual interpretation score of these nodes on EARL1. The horizontal line in the middle of the box represents the median, the colored box represents the interquartile range, whiskers represent 95% confidence intervals, and dots represent outliers. Statistically significant differences (*p* < 0.05) between EARL1 and EARL2 per nodal score group are indicated by * on the x-axis

All 175 delineated lymph nodes were scored independently by two nuclear medicine physicians by means of visual interpretation of nodal [^18^F]FDG uptake. The interobserver agreement was high, with a kappa of 0.84 using dichotomized scores (1–2 vs. 3–4) and kappa 0.73 using scores 1–2 vs. 3 vs. 4. Figure 3 demonstrates the maximum [^18^F]FDG uptake (i.e., SUV_max_ and SUR_max_) stratified by the visual interpretation score of lymph nodes on EARL1 imaging. All nodes that were visually scored "3 – probably malignant" on EARL1 had a SUR_max_ ≥ 1.5 and ≥ 1.6 on EARL1 and EARL2, respectively. All nodes that were scored "4 – definitely malignant" on EARL1 had a SUR_max_ of ≥ 2.2 on EARL1 and ≥ 2.7 on EARL2. For lymph nodes visually scored “2—probably benign” on EARL1, 38% (11/29) was upstaged to score “3—probably malignant” on EARL2 (Table 5). For lymph nodes visually scored “3—probably malignant” on EARL1, 29% (6/21) was upstaged to score “4—definitely malignant” on EARL2. As a result, the N-classification changed in 18% (6/33) of the patients, with consequences for radiotherapy target volume in 24% (8/33) of the patients (i.e., additional lymph nodes irradiated with a high dose). There were no lymph nodes downstaged on EARL2 that were scored 3 or 4 on EARL1.

**Table 5:** Visual scores of lymph nodes on EARL1 and EARL2

EARL1 (n)	EARL2 (n)				Total
	Score 1	Score 2	Score 3	Score 4	
Score 1	*75 (81%)*	17 (18%)	1 (1%)	0	93 (100%)
Score 2	3 (10%)	*15 (52%)*	**11 (38%)**	0	29 (100%)
Score 3	0	0	*15 (71%)*	**6 (29%)**	21 (100%)
Score 4	0	0	0	*32 (100%)*	32 (100%)
Total	78	32	27	38	175

Bold values represent lymph nodes that were upstaged from score “2—probably benign” on EARL1 to score “3—probably malignant” on EARL2, or from score “3—probably malignant” on EARL1 to score “4—definitely malignant” on EARL2. Italic values represent lymph nodes that were neither upstaged nor downstaged on EARL2 compared to EARL1

## Discussion

This study demonstrates a significant increase in SUV_max_ (16.5%) and SUR_max_ (9.6%) of primary tumor and lymph nodes on EARL2 reconstructed imaging compared to EARL1 in patients with HNSCC. Absolute differences in volume and spatial overlap of MTVs were small between EARL1 and EARL2 reconstructed images, irrespective of the segmentation method used. Relative differences in MTVs were small using the adaptive threshold method and larger when using static SUV or SUR thresholds. Moreover, as a result of a higher SUV_max_ on EARL2 reconstructed images, more lymph nodes were likely to be scored as (probably) malignant with visual interpretation. This would have had consequences for the N-classification in 18% (6/33) and affecting radiation treatment in 24% (8/33) of the patients. These observations in a cohort with head and neck cancer patients are in line with the results of several previous phantom and clinical studies in other tumor sites, such as lymphoma and non-small cell lung cancer [6, 8, 9].

The SUV_max_ was on average 16.5% higher on EARL2 compared to EARL1 reconstructed images, with a strong correlation for both SUV_max_ and SUR_max_ between EARL1 and EARL2. In patients with lymphoma and non-small cell lung cancer, Kaalep et al. found that SUV_max_ on EARL2 was on average 34% higher compared to EARL1 [8]. In line with our study, the largest differences in maximum [^18^F]FDG uptake between EARL1 and EARL2 were observed in smaller lesions. This can be explained by the better resolution of EARL2 reconstructed images and thus reducing the partial volume effect [5, 6]. The current study demonstrates a smaller but still significant increase in the maximum [^18^F]FDG uptake on EARL2 when using a target to background ratio (SUR_max_) compared to SUV_max_. Few other studies demonstrate that the use of tumor-to-liver ratios also do not completely mitigate the effect of different EARL reconstructions [8, 19].

Absolute differences of primary tumor MTVs between EARL1 and EARL2 were small (< 1.0 cm^3^), independent of the segmentation method used. This is clinically important for radiation dose escalation to MTVs within the primary tumor volume based on [^18^F]FDG PET imaging. Although absolute differences in MTVs between both EARL reconstructions were small, the differences were still statistically significant for most segmentation methods (7/9). In contrast to static SUV or SUR thresholds, we observed that MTVs were smaller on EARL2 using relative threshold methods (i.e., MAX40% & MAX50%). This is in line with the results reported by Kaalep et al. [8]. However, they reported a median difference in MTV on EARL2 of -27% compared to EARL1 with the MAX41% segmentation method, compared to only -7.5% in our study [8]. Recently Ferrandez et al. calculated changes in MTV between EARL1 and EARL2 in 56 lymphoma lesions [20]. For the MAX41% and SUV2.5 method MTVs decreased with 27% and 4%, respectively. The smaller differences in MTVs observed between EARL1 and EARL2 in the current study may result from the use of time-of-flight and point spread function in both EARL reconstructions while this was not the case in the other studies. Therefore, differences in MTVs were most likely the result of different pixel and filter sizes only. Finally, patients with lymphoma and non-small cell lung cancer generally have larger tumor volumes than patients with HNSCC. Although absolute tumor volumes were not reported by Kaalep et al., and thus cannot be compared to the current data, this could potentially have contributed to the different findings in our study.

In literature several post-acquisition harmonization methods have been described to minimize variability in MTVs when using EARL2 vs. EARL1 reconstructed images. Kaalep et al. performed post-filtering of EARL2 reconstructed images with a 6–7 mm Gaussian filter, in order to generate EARL1 compliant quantitative data from EARL2 images [8]. This would obviate the need to perform a EARL1 compliant reconstruction, while both EARL2 and EARL1 images are still available to allow comparison of quantitative data with historic cohorts. Recently Ferrandez et al. investigated the ComBat harmonization method, aiming to align MTVs from EARL1 and EARL2 reconstructed images [20]. This ComBat harmonization resulted in an improved agreement of MTVs from different reconstructions for most segmentation methods. The advantage of ComBat is that it directly applies to quantitative metrics already extracted from the images based on assumptions and estimations of batch effects, without the need to actually have access to the images [21]. A limitation is that the transformation is specific for each type of tissue, tumor, scanner and segmentation method. In a prospective setting, such as in our study, we strongly believe in the importance of upfront harmonization strategies (like EARL) and advise that both EARL1 and EARL2 reconstructed images are acquired for each patient. This allows for a direct comparison of quantitative [^18^F]FDG uptake metrics on both images, next to morphological features of the lesion. However, in a retrospective setting, post-acquisition harmonization methods such as ComBat and post-filtering can be useful when comparing quantitative metrics based on the latest EARL protocol (e.g. EARL2 or in the future EARL3) with historic cohorts.

For the majority of segmentation methods (8/9), CE values ranged between 0.10 and 0.20, indicating a good spatial overlap of MTVs on both EARL images. This is especially important in radiation treatment planning, as false-negative and false-positive volumes may impact tumor control probability or treatment induced toxicity.

For TLG, differences between EARL1 and EARL2 were also dependent on the segmentation method used. Relative differences were smallest using the MAX40% method and larger using static SUV thresholds. Kaalep et al. reported a median relative difference in TLG on EARL2 of 23% compared to EARL1 with a static threshold of SUV4 [8]. For the MAX41% method the TLG on EARL2 decreased with only 2%. These results are comparable to the findings in our study when using SUV3.5/4.5 and MAX40% thresholds. As TLG reflects the total [^18^F]FDG accumulation in the lesion, which obviously should be equal for both EARL reconstructions, it should be less sensitive to different reconstruction methods and lesion size compared with SUV_max_ [8, 22, 23]. Based on our results, the MAX40% method may be a good candidate for estimating the TLG because the differences between EARL1 and EARL2 were small. This is relevant because there is an increasing interest in TLG in literature as several studies reported that changes in TLG during treatment are predictive for loco-regional control and overall survival in patients with HNSCC [24, 25].

Our analysis demonstrated that quantitative visual evaluation of cervical lymph nodes on EARL2 compared to EARL1 would have changed the N-classification in 18% and affected radiation treatment in 24% of the patients. Similarly, Ly et al. showed that in 52 lymphoma patients EARL2 versus EARL1 reconstructions led to an upgrade in Deauville score in 18 patients (33%), resulting in a treatment intensification in 5 patients (9%) [9]. As such, caution is warranted when applying quantitative [^18^F]FDG uptake thresholds, that are based on EARL1 imaging, directly to EARL2 diagnostic imaging as this comes with a risk of upstaging and overtreatment. Therefore, EARL1 based quantitative thresholds should be re-evaluated before being implemented on EARL2 imaging.

## Conclusions

Implementation of the EARL2 reconstruction methods for [^18^F]FDG-PET imaging resulted in a higher SUV_max_ and SUR_max_ in primary tumors and lymph nodes, compared to the EARL1 image reconstruction. Using EARL2 versus EARL1 images for the visual interpretation of lymph nodes led to nodal upstaging and alteration of radiation treatment volumes in a significant amount of patients with HNSCC. Further research is needed to re-evaluate [^18^F]FDG uptake thresholds based on EARL1 before they can be applied on EARL2 imaging.

## Data Availability

The datasets used and/or analyzed during the current study are available from the corresponding author on reasonable request.

## Abbreviations

PET: Positron emission tomography
[^18^F]FDG: Fluor-18-fluorodeoxyglucose
CT: Computed tomography
HNSCC: Head and neck squamous cell carcinoma
EANM: European Association of Nuclear Medicine
EARL: EANM Research Ltd.
SUV: Standardized uptake value
MTV: Metabolic target volume
TLG: Total lesion glycolysis
OSEM: Ordered-subsets expectation–maximization
SUR: Standardized uptake ratio
CE: Classification errors
CI: Confidence interval
IQR: Interquartile range
